# Bortezomib, Lenalidomide and Dexamethasone Combination Induced Complete Remission in Relapsed/Refractory Plasmablastic Lymphoma: Case Report of a Potential Novel Treatment Approach

**DOI:** 10.3390/curroncol29070399

**Published:** 2022-07-18

**Authors:** Waleed Sabry, Yue Wu, Shruthi Ganeshappa Kodad

**Affiliations:** 1 Saskatoon Cancer Centre, College of Medicine, University of Saskatchewan, 20 Campus Drive, Saskatoon, SK S7N 4H4, Canada; 2 Department of Pathology and Laboratory Medicine, College of Medicine, University of Saskatchewan, Saskatoon, SK S7N 0W8, Canada; yue.wu@saskhealthauthority.ca

**Keywords:** plasmablastic lymphoma (PBL), targeted therapy, bortezomib

## Abstract

Plasmablastic lymphoma is a rare subtype of large B-cell lymphoma characterised by an aggressive clinical course with frequent relapses and refractoriness to chemotherapy. It is usually associated with HIV, however, it can also be seen in immunocompetent patients. It has distinct pathological characteristics, such as plasmablastic morphology and lack of CD20 expression. These characteristics pose a clinical and pathological challenge. There is no standard of care established in this entity. In this case report, we described a novel bortezomib-based plasma cell targeted regimen in a HIV-negative patient refractory to chemotherapy.

## 1. Introduction

Plasmablastic lymphoma (PBL) is a rare, aggressive type of large B-cell lymphoma characterised by a CD20 negative phenotype. It was initially described in HIV-positive patients, comprising 2% of all HIV-associated lymphomas. Later on, it was identified in other patients, including HIV-negative immunocompromised, post-transplant, and immunocompetent patients, with no actual incidence. The prognosis is dismal in HIV-positive and negative patients [1,2]. Diagnosis and treatment of PBL are challenging due to the disease’s rarity, and the mixed and overlapped features between lymphoma and myeloma. PBLs have overlapping morphologic and immunophenotypic characteristics of aggressive large B-cell lymphomas; however, pan-B-cell markers such as CD20 and PAX5 are usually absent, whereas features of plasmacytic differentiation including CD138, CD38, and MUM1/IRF4 are positive [3,4]. It is very difficult to design controlled trials for rare diseases, such as PBL, and the current knowledge depends only on information obtained from small case series and case reports. In the absence of a standard of care, different chemotherapy combinations have been used with no success to treat this disease, which runs a course of short-lived remissions and refractoriness. Therefore, there is an un-met need to identify novel therapeutic approaches to treat PBL, including targeted therapy. We herein report a case of a transplant-ineligible patient who achieved complete remission with a myeloma-like regimen combining bortezomib, lenalidomide, and dexamethasone for a relapsed/refractory PBL.

## 2. Case Presentation

A 59-year-old male presented in May 2019 to the emergency room with a two-week history of left frontal sinus pain, swelling, and pain below the left eye. He had a significant history of congestive heart failure, atrial fibrillation, eosinophilic granulomatosis with polyangiitis (Churg–Strauss syndrome), and chronic sinusitis with multiple polyps. He was treated for an extended period with steroids, and was started on azathioprine shortly before presentation. His symptoms progressed over two weeks with discomfort and difficulty moving his left eye together with proptosis. There was no cranial nerve involvement, vision disturbances, or other neurological deficits. A CT scan was performed on 10 May 2019, which identified extensive soft tissue density in the left paranasal sinuses and extension into the left orbit and left anterior cranial fossa. There was bony invasion and destruction of the left anterior cranial fossa, and a small amount of left frontal extra-axial tumour extension. He was given another oral prednisone 50 mg daily-course and IV antibiotics. He was then referred to an ENT specialist. On 26 June 2019, he underwent an endoscopic excision of the left sinonasal mass, left anterior, posterior ethmoidectomies, left middle meatal antrostomy, removal of the middle turbinate, left intranasal sphenoidotomy, and left frontal sinusotomy.

**Figure 1:**
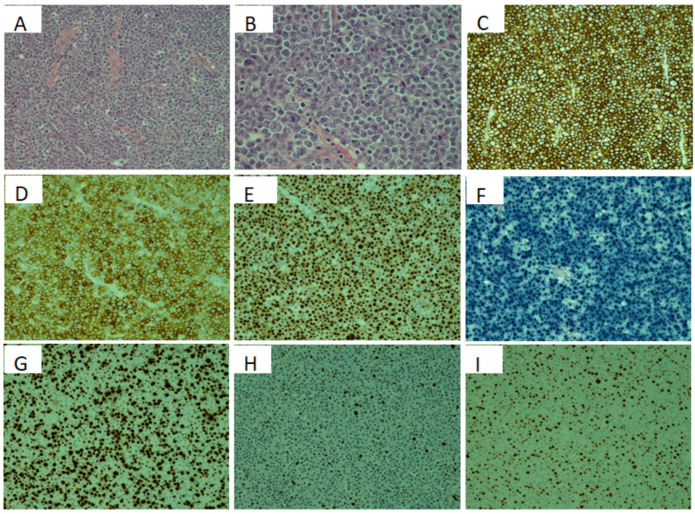
Histopathology pictures of left sinonasal mass: (**A**,**B**) Hematoxylin and Eosin stained slides (200× and 400×, respectively) of this mass lesion. (**C**) CD138 IHC shows diffuse positivity. (**D**) CD38 IHC shows diffuse positivity. (**E**) MUM1 IHC shows diffuse positivity. (**F**) EBV in-situ hybridization (EBER) shows diffuse RNA positivity. (**G**) Ki67 IHC shows 70% proliferation index. (**H**) P53 IHC shows rare positivity (5%). (**I**) *c-MYC* IHC shows nearly 50% positive cells.

Investigations: The histopathology showed sheets of predominantly large atypical lymphoid cells displaying prominent nucleoli resembling plasmablasts, accompanied by abundant mitotic bodies and apoptotic figures. Tumor cells were diffusely positive for CD138, CD38, CD79a, MUMI, kappa light-chain restriction, CD56, and EBV-RNA (demonstrated by EBV is-situ hybridization) (Figure 1). They were negative for pankeratin, CD20, PAX5, CD117, cyclin D1, CD30, HHV8, and T cell markers. The proliferation index (Ki67) of tumor cells is 70%. The histology features support a diagnosis of plasmablastic lymphoma. Approximately, 50% of the lymphoma cells show positivity with *c-MYC* by immunohistochemistry (IHC). P53 IHC shows rare positivity in those extremely large cells (5% positivity). FISH analyses were performed on the paraffin block of this sinonasal mass specimen. *c-MYC* gene rearrangement is negative. Multiple myeloma FISH panels (1p32.3/1q21 CDKN2C/CKS1B, del(13q)/13q14/13q34 and del(17p)TP53) are negative for abnormalities.

**Figure 2:**
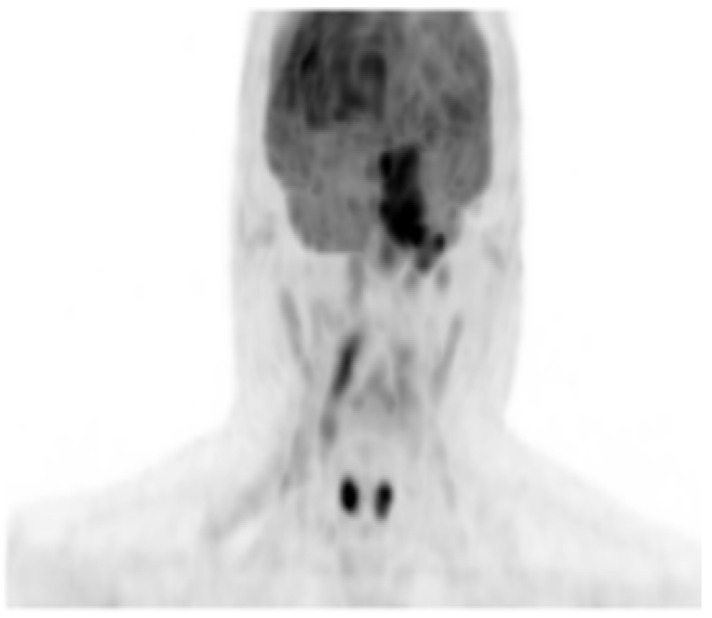
PET scan image at diagnosis in 7 July 2019.

The staging CT and PET scan did not show any evidence of lymphoma elsewhere, including a negative bone marrow biopsy (Figure 2). The PET scan confirmed an intensely FDG-avid mass lesion in the left maxillary sinus, ethmoid air cells, bilateral frontal sinuses, left orbit, and left nasal cavity with a maximal SUV of 10.2 compatible with his sinonasal lymphoma. CSF analysis was negative for malignant cells. Beta-2-Microglobulin and lactate dehydrogenase (LDJ) were both within the normal range at diagnosis.

**Figure 3:**
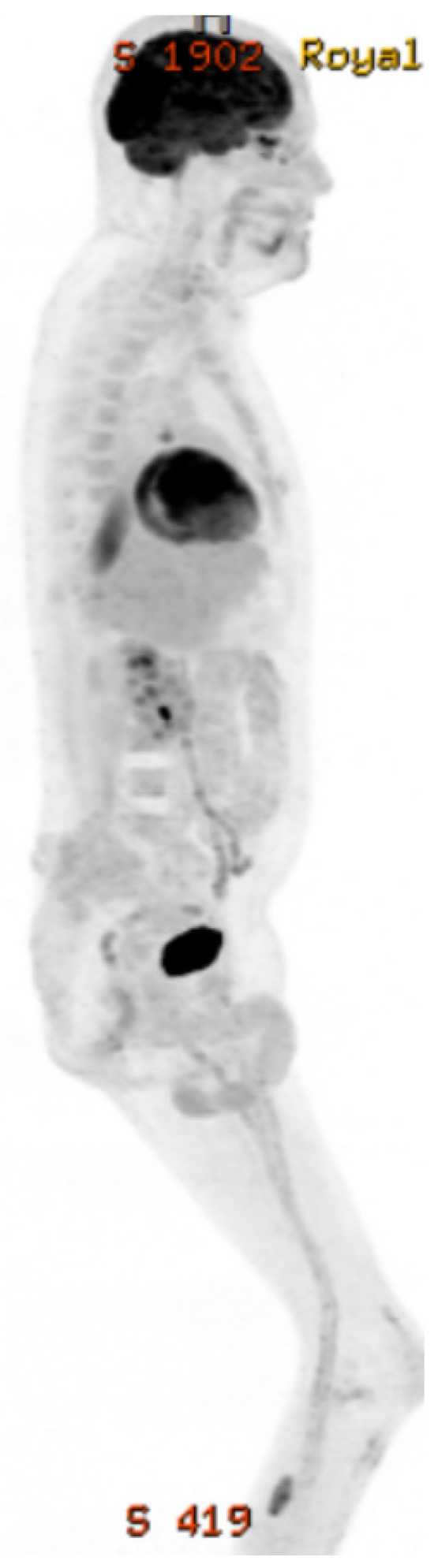
Evidence of relapse on the PET scan performed on December 2019.

Treatment and outcomes: Due to the history of congestive cardiac failure, we opted not to use doxorubicin in the CHOP regimen and replaced it with etoposide. He was started on combination chemotherapy using cyclophosphamide, etoposide, oncovin, and prednisone (CEOP) regimen using the following doses: cyclophosphamide 750 mg/m^2^ IV day 1, etoposide 50 mg/m^2^ IV day 1, etoposide 100 mg/m^2^ PO daily on days 2 and 3, vincristine 1.4 mg/m^2^ IV day 1 and prednisone 100 mg PO once daily on days 1–5. He also received prophylactic intrathecal methotrexate at a dose of 15 mg. Weekly Rituximab (375 mg/m^2^) for 4 weeks was added due to high titers of EBV according to the PCR test results. Approximately 10 days after chemotherapy, he developed a diminished level of consciousness. A CT brain, identified a low-density extra-axial collection in the right frontal and parietal lobes, and a smaller area in the left frontal lobe in keeping with a subacute or chronic subdural haemorrhage. He underwent a burr hole drainage of the subdural collection which was later identified as empyema at the time of surgery. Post-surgery, he developed a pneumocephalus with mass effect for which he required 3 craniotomies in August 2019. The procedure on 15 August 2019, included a bitemporal craniotomy with a frontal flap, and endoscopic sinus surgery for resection of the sinonasal lymphoma with intracranial extension. This procedure was for clearing of the areas of infection, residual tumour, and repair of a skull-based defect resulting in tension pneumocephalus. This identified residual plasmablastic lymphoma on the left frontal sinus. Closed space cultures grew staphylococcus hominis, prevotella species, and pseudomonas aeruginosa. He was, therefore, followed by the infectious disease team and received IV antibiotics as per sensitivities for approximately 50 days with a slow hospital recovery. A repeat CT brain demonstrated persistence of the residual subdural collection in the right frontal lobe. The patient was accordingly discharged on the home IV program to receive meropenem, vancomycin and fluconazole for four more weeks. The Radiation Oncology team was consulted to attempt to control any disease progression while waiting to resume systemic treatment. He received radiation therapy (RT) directed at the left nasal cavity and surrounding sinuses. A VMAT technique using 6 MV photon beam arcs was used to deliver 4000 cGy in 20 fractions over 4 weeks. RT was well tolerated with no significant complications and was completed on 8 October 2019. The CT brain after completion of IV antibiotics and RT, did not show any residual collection. He went on to receive the 2nd cycle of R-CEOP and high dose methotrexate (3000 mg/m^2^) on day 10. He completed 3rd R-CEOP and was admitted to receive the day 10 high dose methotrexate when he developed persistent left leg pain. An X-ray of the left leg showed osteolytic lesions. An MRI showed two aggressive lesions in the proximal and mid-fibular diaphysis. The biopsy was inconclusive due to scanty tissue. A PET scan confirmed the left fibular metastatic lesions corresponding to the MRI (SUV 8.7). There was also an FDG avid lesion at the right distal humerus (Figure 3).

**Figure 4:**
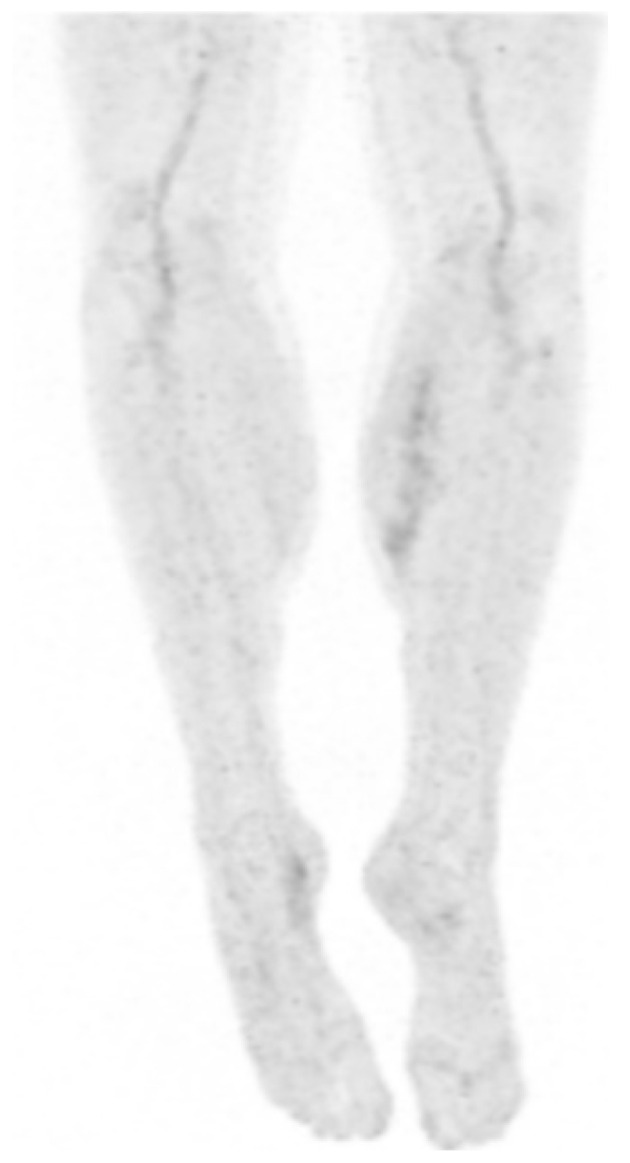
PET in February 2021 CR showing evidence of remission.

**Figure 5:**
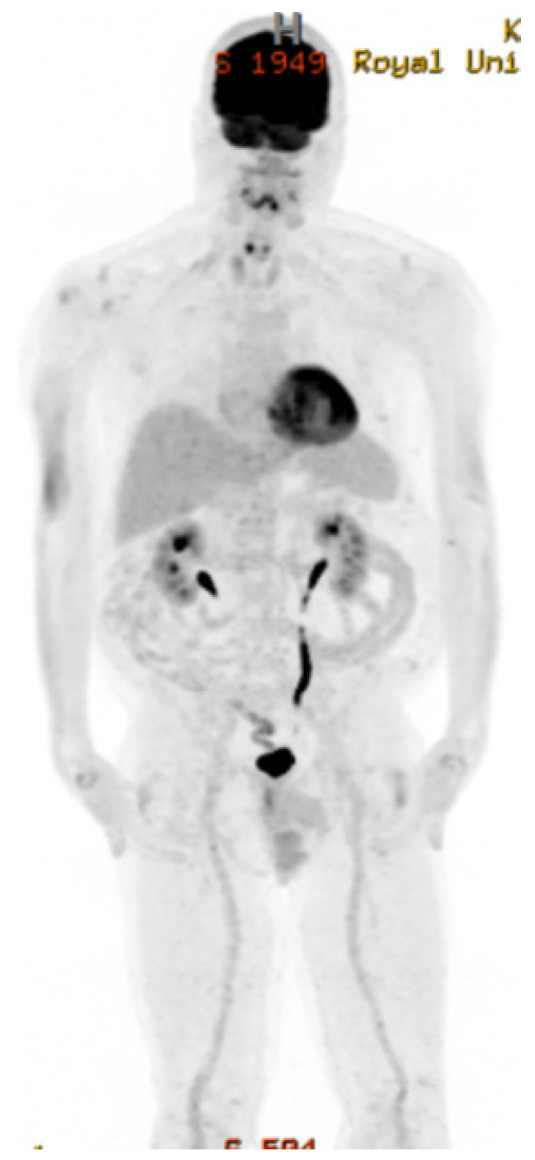
PET in February 2021 showing complete remission.

He was considered to be refractory to first-line treatment and was initiated on lenalidomide 25mg PO days 1–21, bortezomib 1.3 mg/m^2^, subcutaneously on days 1, 8, 15, and 22 and dexamethasone 20 mg PO on days 1, 8, 15, and 22 (RVD) regimen in December 2019. PET CT scan after 2 cycles of RVD showed a complete response without any residual lymphoma. Due to excellent response, we continued the RVD regimen for four more cycles before continuing on lenalidomide maintenance at a dose of 10 mg daily on days 1–21 of a 28-day cycle. A PET CT scan in July 2020 showed FDG activity in the paranasal sinuses however, an MRI confirmed no evidence of recurrence of lymphoma (Figure 4 and Figure 5). He was started on maintenance lenalidomide in August 2020. PET CT scan in February 2021 still showed a left frontal sinus FDG avidity which was less compared to the previous, and an MRI did not show any evidence of disease. He is on regular follow-up with the ENT specialist and local examinations have been normal. PET CT scan was repeated in September 2021, two months before completion of lenalidomide maintenance, and showed a new intensely hypermetabolic pulmonary nodule in the apical segment of the right upper lobe measuring 0.8 cm with an SUV of 4.8, suspicious of primary or metastatic malignancy until proven otherwise. The lesion was too small to biopsy and a dedicated chest CT scan was performed two weeks later showing no residual nodules or masses corresponding with the one on the PET CT scan suggesting that it was most probably of an infectious/inflammatory cause. Lenalidomide maintenance was completed in November 2021 and an MRI of the brain, orbits, and facial region with contrast showed no evidence of residual or recurrent sinonasal tumour. Until the end of May 2022, when this report was being finalised, the patient remained in complete clinical and radiologic remission. 

## 3. Discussion

The optimal chemotherapy combination for the treatment of PBL has never been established due to the rarity of the disease as well as its aggressive nature. CHOP (cyclophosphamide, doxorubicin, vincristine, and prednisone) combination chemotherapy, which is the standard of care in many institutes for treating aggressive lymphomas, does not usually confer a durable remission in those patients [5,6,7,8]. Many experts in the field would consider dose-adjusted EPOCH (etoposide, prednisone, vincristine, cyclophosphamide, and doxorubicin) as a first-line treatment over CHOP given the benefit shown in a meta-analysis of HIV-positive PBL [9]. 

Different regimens to treat aggressive lymphomas, such as hyper-CVAD (hypofractionated cyclophosphamide, vincristine, doxorubicin, and dexamethasone alternating with methotrexate and cytarabine) or the MAGRATH protocol (cyclophosphamide, vincristine, doxorubicin, high-dose methotrexate 9 (CODOX-M)/alternating with ifosfamide, etoposide, and high-dose cytarabine (IVAC) have been tried with minimal ability to identify a standard of care for treating PBL. Depending on the affected sites, central nervous system (CNS) targeted therapy would be considered on a case-by-case basis [10,11,12,13]. Patients with chemosensitive disease achieving a partial or complete remission after the 1st line of treatment should be considered for high-dose chemotherapy and autologous stem cell transplant (ASCT) in 1st remission depending on eligibility [14,15]. Autologous stem cell transplant in relapsed/refractory cases has been used with some success according to a few reports [16,17,18]. Allogeneic stem cell transplantation (allo-SCT) in PBL, on the other hand, does not seem to be very promising and cannot be considered a standard of care [19,20].

In patients with localized disease, radiotherapy alone or combined with chemotherapy may achieve good disease control in some cases. The use of radiation therapy in advanced PBL is usually not considered in the first-line setting but could be entertained in relapsed/refractory patients [21,22,23]. 

Spontaneous regressions have been observed in certain HIV-infected patients after the initiation of active treatment with CAR-T or highly active anti-retroviral therapy [24,25]. An Italian study showed a 3-year OS of 67% in 13 HIV-positive PBL patients treated with CAR-T [16].

Forty to fifty percent of PBL patients may have involvement in the oral cavity with more prevalence in HIV-positive patients who also have more incidence of bone marrow involvement, advanced stage, and B-symptoms. PBL was identified in the post-transplant setting whether in solid organ transplant or hematopoietic stem-cell transplant. A limited number of cases with PBL transforming from other indolent lymphomas, such as CLL/SLL or follicular lymphoma have also been identified [26].

The prognosis of HIV-positive patients with PBL is very poor and the median overall survival (OS) in the literature ranged between 7 and 15 months with the 5-year OS <25% in most series [15,27,28,29] although, a 5-year OS of 40% was reported in a single institution with the use of hyper-CVAD-MA regimen [30]. 

The prognosis may be slightly better in HIV-negative PBL patients, especially in those who get consolidation with autologous HSCT in CR1. The use of immunosuppression among HIV-negative patients may confer, however, a worse outcome [31].

The International Prognostic Index (IPI) scoring system is the most commonly used risk stratification tool for aggressive lymphomas, and it includes age, performance status, lactate dehydrogenase (LDH) levels, number of extranodal sites, and clinical-stage to prognosticate survival [32]. Age and LDH levels were associated with adverse outcomes in one study [33] and did not seem to be of value in another [15]. 

The value of EBV-related antigens expression in the prognosis of PBL is still not well defined. EBV-positivity could pose a diagnostic dilemma as morphologic and phenotypic features of PBL may overlap with a clinically indolent EBV-positive extramedullary plasmacytoma. In our patient, morphologically tumour cells are large in size and show large prominent nucleoli associated with increased mitotic figures and apoptotic bodies. The tumour cells morphologically are thus consistent with large lymphoma cells such as abnormal plasmablasts. In conjunction with the nasal cavity location of this mass lesion, the patient’s iatrogenic immunosuppressed state (due to longstanding steroid treatment for his eosinophilic granulomatosis with polyangiitis) and strong EBV-positivity in the tumour cells, the overall findings support the diagnosis of PBL. In contrast, EBV-positive plasmacytoma usually shows bland mature plasma cell morphology, and is presented in immunocompetent patients [34]. Some studies have shown that EBV expression does not confer a worse outcome in HIV-positive PBL [33,35,36].

Patients with *MYC* gene rearrangements (gains or translocations) had shorter OS, according to a systematic review assessing 57 patients with PBL, compared to patients with a normal *MYC* [28]. These rearrangements were even associated with a 6-fold increased risk of death from any cause in HIV-positive patients with PBL [15]. *MYC* gene rearrangement was reported in 50% of plasmablastic lymphoma cases. It was also reported in some plasmacytoma/multiple myeloma cases associated with high proliferation index Ki67 [28]. This sinonasal mass lesion is negative for *c-MYC* gene rearrangement. The less aggressive clinical course of this PBL could be attributed to its lack of *c-MYC* gene rearrangement.

A series of studies showed a worse outcome in patients with high Ki-67 expression, >80% [15,35,36,37]. While a smaller study showed a negative prognostic value for Ki-67 expression [33].

In HIV-positive PBL, CD4^+^ counts <0.2 × 10^9^/L were not associated with worse OS [15,16], but appeared to be associated with shorter progression-free survival time [15,33].

### 3.1. Plasma Cell-Targeted Treatment Success

#### 3.1.1. Bortezomib

Bortezomib is a known proteasome inhibitor used mainly for the treatment of multiple myeloma (MM), but has been also tried with some success in some types of lymphoma, especially in non-germinal B-cell centres, such as DLBCL, lymphoplasmacytic lymphoma/Waldenstrom macroglobulinemia, and mantle cell lymphoma [38,39,40]. In their review, Guerrero-Garcia et al. reported the use of bortezomib in a series of 21 patients, 11 as a first-line, and 10 with relapsed disease. In the frontline setting with bortezomib-based chemotherapy, out of the 11 patients, 8 achieved CR and 1 achieved PR. With bortezomib alone, 2 patients out of the 11 achieved PR. In the relapsed cohort, with bortezomib-based chemotherapy 5 out of 10 patients were able to achieve PR and 1 was in CR. Comparatively, with bortezomib alone, 3 out of 10 patients achieved PR and 1 had progressive disease [41].

When combined with CHOP and administered as frontline, bortezomib achieved CR in three HIV-associated PBL patients. Two of them were alive 14 and 22 months after receiving the combination of bortezomib and CHOP [42].

Bortezomib has been also used in combination with other chemotherapy protocols in the relapsed setting such as THP-COP (pirarubicin, cyclophosphamide, vincristine, and prednisone), ICE (Ifosfamide, carboplatin, and etoposide), ESHAP (etoposide, high-dose prednisolone, high-dose cytarabine, and platinum), bendamustine, rituximab, and DT-PACE with a CR rate of 16%, and a PR rate of 84% [43]. In 2018, Dittus et al. reported a series of 8 patients with a CR rate of 87.5%, a 2-year FS, and an OS of 50% [44].

In the same year, Castillo et al. reported another series of 16 patients with a CR rate of 94%, with an OS of 63% at five years with two patients receiving ASCT as consolidation [43].

#### 3.1.2. Lenalidomide

Lenalidomide, as an immunomodulatory agent, has been used with great success in treating patients with MM. It has also been tried alone or in combination with chemotherapy in the treatment of subtypes of diffuse large-B-cell lymphoma and showed some efficacy in the non-germinal centre, DLBCL [45]. There was a report of two cases showing a good response, but short-lived with using single-agent lenalidomide in refractory PBL [46,47]. It showed success also when combined with CHOP or cyclophosphamide dexamethasone [48,49]. Ando et al. used bortezomib to treat chemo refractory PBL patients, which provided a clinical response, but was discontinued due to peripheral neuropathy. The patient then received a combination of lenalidomide and dexamethasone for over two years with continuing partial response [50].

A patient with PBL refractory to mini-CHOP as a first-line achieved complete remission after treatment using tislelizumab, a checkpoint inhibitor, and in combination with lenalidomide [51]. Marrero et al. treated a patient with relapsed PBL with parotid involvement using a combination of lenalidomide and bortezomib. The patient received two cycles of the protocol only which was discontinued due to developing pancreatitis attributed to bortezomib. PET CT scan performed after the two cycles showed no evidence of disease and the patient remained in complete remission for at least 12 months from the onset of the salvage therapy [52].

#### 3.1.3. Brentuximab

Brentuximab, an anti-CD 30 agent, was used in a heavily pre-treated patient, with positive expression of CD 30 on lymphoma cells. Despite being refractory to multiple chemotherapy regimens and radiation, the patient achieved an objective response after receiving brentuximab indicating the potential use of this agent in CD 30 positive PBL [53].

#### 3.1.4. Daratumumab

Given that PBL universally expresses CD 38, Dittus et al. recently described four patients with relapsed PBL who received a combination of daratumumab, ifosfamide, carboplatin, and etoposide (D-ICE). All patients achieved a CR after 3–4 cycles of therapy [54]. One case report evaluated daratumumab with bortezomib and lenalidomide in a patient with relapsed PBL who achieved a PR, and proceeded to an allogeneic stem cell transplant after [55].

Daratumumab was also combined with EPOCH as a frontline therapy for three patients and with lenalidomide, dexamethasone, and doxorubicin in one patient with relapsed disease. All patients achieved CR with durable remission for at least 15 months [56].

A prospective trial evaluating daratumumab, bortezomib, and lenalidomide is currently active (NCT04915248). Another prospective study conducted by the AIDS Malignancy Consortium is also evaluating daratumumab–EPOCH in the frontline setting (NCT04139304) [57].

**Figure 6:**
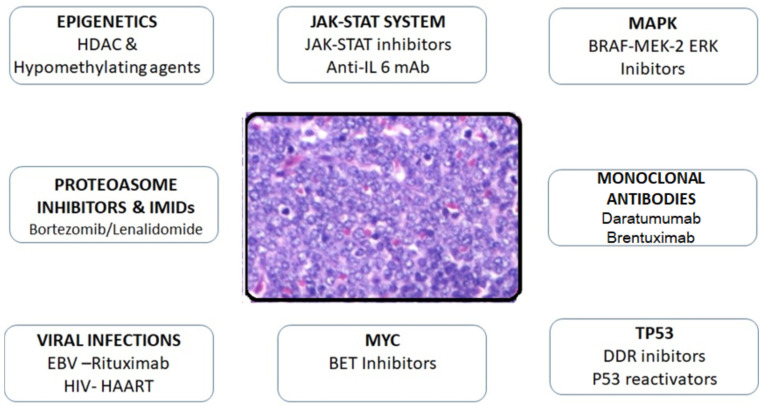
Different therapeutic options and targets in PBL, Modified from Pileri SA, Mazzara S, and Derenzini E [58]. Abbreviations: (HDAC: histone deacetylase, BRAF: B-Raf proto-oncogene, MEK: mitogen-activated protein kinase, ERK: mitogen-activated protein kinase, DDR: DNA damage response pathway, BET: bromodomain and extra terminal protein, HAART: highly active anti-retroviral therapy).

Figure 6 illustrates the different targets and therapeutic options for the management of PBL.

## 4. Conclusions

Plasmablastic lymphoma is a challenging disease, both in terms of its diagnosis and treatment. Most long-term survivors were fit to undergo autologous stem cell transplants as consolidation following response to combination chemotherapy. Myeloma-based regimens including proteasome inhibitors, immunomodulators, and targeted therapy are paving the way for better results. Our patient was not only transplant-ineligible due to severe congestive cardiac failure, but also was not a candidate to receive an anthracycline-based regimen as a frontline. The sinonasal mass did respond to the CEOP regimen, however, due to the infections leading to subdural empyema, systemic therapy was interrupted with long gaps. With the relapse of the disease, we were able to achieve a complete response with the RVD regimen. Not only did he maintain this response with lenalidomide, but also did not develop any significant toxicities. Further studies are needed to investigate the benefits of myeloma-based therapies in the firstline setting for patients who are not candidates for conventional chemotherapy-based regimens or autologous transplants.

## Data Availability

Not applicable.
